# Peripheral mononuclear blood cell apheresis in a preclinical ovine model

**DOI:** 10.1186/s12917-018-1332-4

**Published:** 2018-02-13

**Authors:** Helen Lydon, Roger Brooks, Andrew McCaskie, Frances Henson

**Affiliations:** 1 Department of Surgery, University of Cambridge, Hill’s Road, Cambridge, UK; 2 Department of Veterinary Medicine, University of Cambridge, Madingley Road, Cambridge, UK

**Keywords:** G-CSF, Apheresis, Haematopoetic stem cells, CD34, Ovine, Sheep

## Abstract

**Background:**

Recent research has demonstrated that circulating peripheral blood mononuclear fractions (PBMC) containing haematopoietic stem (HSC)/progenitor cells have the potential to play a crucial role in regenerative medicine strategies. Work in our laboratory has shown that a peripheral blood mononuclear cell fraction (PBMC) enhances cartilage repair in an osteochondral defect model in sheep and has a significant effect on cells in the joint niche. In order to obtain PBMC rich blood containing HSCs for further studies, we have performed, for the first time, apheresis on adult sheep.

**Results:**

Subcutaneous granulocyte-colony stimulating factor (G-CSF) was used to mobilise white blood cells and continual flow apheresis was performed on 8 sheep under general anaesthetic. There were no observable side effects, although a marked tendency for blood clotting during the procedure was noted. The administration of G-CSF for 3 days increased the white blood cell (WBC) count in the peripheral blood from to 6.7 ± 2.1 × 10^6^/ml to 16.1 ± 5.0 × 10^6^/ml. Following apheresis, the WBC numbers in the apheretic product increased to 38.5 ± 27.6 × 10^6^/ml, comprised of a significant increase in neutrophils and PBMC (from 5.25 ± 1.8 × 10^6^/ml following G-CSF stimulation to 27.5 5 ± 27.6 × 10^6^/ml). There was a mean of 2.1% CD34 + ve cells and 95.5% CD45 + ve cells in the apheretic product.

**Conclusions:**

This study describes the administration of G-CSF and subsequent apheresis in adult sheep. The technique is safe when performed as described with no observable side effects. The technique permits collection of an increased WBC fraction containing neutrophils and PBMC in adult sheep. This apheretic product contains CD34 + ve cells, representing an HSC/progenitor population for use in *in vivo* and *in vitro* experiments.

## Background

The repair of articular cartilage defects remains challenging and all currently available methods have their disadvantages. In addition to autologous chondrocytes, a number of cell types have been proposed as stimulators or contributors to repair either alone or in combination. We have described the successful use of a peripheral blood mononuclear cell fraction (PBMC) to enhance cartilage repair in an ovine osteochondral defect model [1]. This study showed that isolated PBMC had effects on migration and differentiation of both chondrocytes and mesenchymal stromal cells (MSC) [2, 3]. PBMC populations contain a number of cell types, including peripheral haematopoietic stem cells (HSC) [4], derived from the bone marrow. CD34 + ve HSC are widely used in regenerative medicine to treat malignant and non malignant haematopoetic disorders [5] and, increasingly, are being used in regenerative medicine strategies to treat disorders of other body systems [6]. Traditionally HSC were harvested directly from the bone marrow for bone marrow transplantation; however, automated retrieval of a peripheral blood progenitor cell (PBPC) fraction for this purpose using an apheresis procedure is now standard clinical practice [7]. The technique is based on the mobilisation of bone marrow cells into the peripheral blood by the administration of exogenous granulocyte colony stimulating factor (G-CSF) [8]. Many studies have described the use of apheresis to collect G-CSF stimulated PBMC in man and apheresis has been described in a number of animal species including dogs [9, 10], monkeys [11] and swine [12].

In order to continue investigating the effect of PBMC and PBPC on the healing of joint surface defects in experimental ovine models, we have performed apheresis in adult sheep to provide concentrated populations of peripheral blood cells for in vitro and in vivo research. In this paper we describe the use of G-CSF to stimulate the bone marrow niche, the method of apheresis in sheep and the cell types harvested.

## Methods

### Animals

Eight 3 year-old (adult) healthy Welsh Mountain female sheep weighing 40-42 kg underwent apheresis. The animals were purchased from a Licenced Supplier specifically for this study. All animal procedures were carried out in accordance with the regulations as set out in the Animal [Scientific Procedures] Act 1986. This study received approval from both the Animal Welfare and Ethical Review Board, Cambridge University and the UK Home Office (Project Licence number 70/8165).

### Donor preparation

Animals were treated with rhG-CSF (Neupogen, Amgen) 5 mg/kg subcutaneously once a day for 3 days prior to apheresis with the final dose being administered 2 h before apheresis commenced [13]. Blood samples collected into EDTA were obtained prior to commencing rhG-CSF administration and immediately prior to apheresis and complete blood counts (CBC) were performed to establish haematological values using an automated haematology analyser.

### Apheresis

A 20 g intravenous catheter was placed into the cephalic vein and anaesthesia induced with a single intravenous bolus of 3 mg/kg alfaxalone (Alfaxan, Jurox). This catheter was secured and subsequently used as the fluid return portal during apheresis. Sheep were intubated and maintained on isofluorane. Animals were placed in dorsal recumbency and jugular venous access obtained via the integral cannula of the closed system apheresis tubing kit. At the completion of apheresis, the jugular cannula and cephalic catheter were removed and the animals were allowed to recover normally, prior to being replaced into their pens.

Apheresis was performed using a Cobe Spectra (Terumo-BCT) with a disposable double needle closed system apheresis tubing kit. The tubing kit and collection chambers were primed with 50 ml acid-citrate-dextrose A (ACD-A, Biomet, USA) anticoagulant and 0.9% saline. PBPC collections were performed on the basis of individual data (body weight and haematocrit). This data, fed into the CobeSpectra at the start of the procedure, automatically established each animal’s total blood volume, the optimal and maximal pump rates and anticoagulant flow rate. Manually varying the plasma pump speed allowed adjustments of the collection product colour and flow rate. A colourgram was used to verify collection of PBMC products.

Throughout apheresis, the body temperature of the animals was monitored to ensure that the temperature remained within normal limits (38.3–39.8). Animals were positioned on a heat retaining mat to reduce heat loss onto the underlying surface and maintain temperature in the physiological range; also, animals were covered with blankets to retain body heat. To avoid ACDA-A solution induced hypocalcaemia, 40 ml of a 10% calcium gluconate solution was administered either subcutaneously during apheresis (*n* = 4) or mixed with blood and returned intravenously throughout the procedure via the cephalic vein (*n* = 3). Apheresis was stopped when 35 ml apheretic product and 30 ml plasma had been collected. Once the flow-back was completed the animal was allowed to recover.

### Apheretic product analysis

A CBC was made of the apheretic product and flow cytometry was used to quantify CD34 and CD45 positive cells in the apheretic samples. Cells were stained with mouse anti-sheep CD45 FITC (0.001 mg mL^−1^; MCA2220F, AbD Serotec, UK) and mouse anti-sheep CD34 (0.005 mg mL^−1^; (AM32448PU-N, Acris/Origene Europe, Germany) [14] conjugated to FITC using FITC Fast Conjugation Kit (Abcam, UK) in staining buffer. Cells were also stained with an equivalent concentration of IgG controls: mouse IgG1-FITC (MCA928F, AbD Serotec, UK) and mouse IgG2a FITC (ab81197, Abcam, UK). Dead cells were excluded based on fluorescence using a viability stain (AquaZombie, Biolegend). Cellular staining was measured using a BD LSR-Fortessa with BD FACSDiva software. Data was analysed using Beckman Coulter Kaluza software (v1.2).

### Statistical analysis

Results are expressed as mean ± standard deviation. Comparisons between cell numbers at different experimental points in the animals was determined by one way ANOVA using Graph Pad prism. A *p* value of < 0.05 was considered statistically significant.

## Results

### Effects of rhG-CSF treatment

No obvious signs of any adverse effects that have been reported in humans [15] were observed in the sheep i.e. there was no allergic reaction, lameness reflecting bone pain or reduction in food or water intake.

**Fig. 1:**
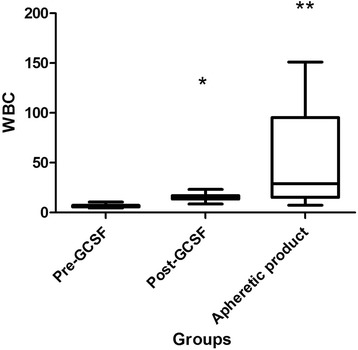
Total white blood cell (WBC) numbers in whole blood before (Pre-GCSF) (*n* = 8) and after administration of G-CSF (Post-GCSF) (*n* = 8) and in the apheretic product (*n* = 7). *Significantly different from Pre-GCSF value, **Significantly different from Pre- and Post- GCSF values. *P* < 0.05. Values ×10^6^/ml blood

**Fig. 2:**
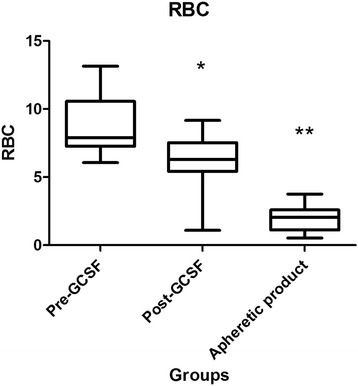
Total red blood cell (RBC) numbers in whole blood before (Pre-GCSF) (*n* = 8) and after administration of G-CSF (Post-GCSF) (*n* = 8) and in the apheretic product (*n* = 7). *Significantly different from Pre-GCSF value, **Significantly different from Pre- and Post- GCSF values. *P* < 0.05. Values ×10^6^/ml blood

CBC were performed in peripheral blood before G-CSF administration and immediately prior to apheresis. Prior to G-CSF administration, the mean WBC concentration was 6.74 ± 2.06 × 10^6^/ml. Following the administration of G-CSF the mean WBC concentration was 16.1 ± 5.0 × 10^6^/ml, a statistically significant 238% increase (Fig. 1). Mean red blood cell (RBC) concentrations prior to G-CSF administration were 7.7 ± 1.7 × 10^6^/ml, with RBC concentrations post-G-CSF administration significantly decreasing to 6.4 ± 1.6 × 10^6^/ml (Fig. 2).

**Fig. 3:**
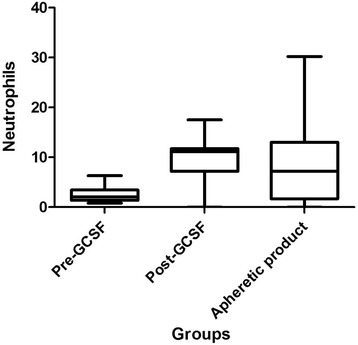
Numbers of neutrophils in in whole blood before (Pre-GCSF) (*n* = 8) and after administration of G-CSF (Post-GCSF) (*n* = 8) and in the apheretic product (*n* = 7)

Individual WBC types were quantified to identify which cell types were being stimulated by G-CSF. Neutrophils and PBMC (lymphocytes + monocytes) were significantly increased following G-CSF treatment, no significant alteration was seen in the granulocyte population (eosinophils + basophils). Neutrophils increased from 3.1 ± 2 × 10^6^/ml pre-treatment to 10.5 ± 4.2 × 10^6^/ml post-treatment, whilst PBMC increased from 3.4 ± 0.6 pre-treatment to 5.2 ± 1.8 post-treatment. (Fig. 3).

One animal did not apparently respond to the administration of G-CSF. In this animal, the pre-G-CSF WBC count was 7.10 × 10^6^/ml but, following the administration of G-CSF, the WBC count had fallen to 3.9 × 10^6^/ml.

### Apheresis procedure

Seven of the eight animals completed the apheresis. In one sheep, rapid and repeated clotting of venous blood within the cannula and tubing at the start of apheresis necessitated abandoning the procedure. In addition, 2 further animals produced blood clots in the cannula and/or tubing immediately upon starting blood withdrawal by the Cobe Spectra. In these animals, replacement catheters and heparin saline flushes were required to continue the apheresis procedure in the initial stages. The mean time required for collection in the 7 sheep was 93 min ± 12 min to obtain 35 ml apheresis product and 30 ml plasma.

### Effects of apheresis

Hypothermia was present in 1/7 sheep upon cessation of the procedure. This animal was immediately covered with blankets and placed into a warmed recovery area until stable. No signs of hypocalcaemia (licking, agitation or tremors in the absence of low body temperature) were detected in any sheep during the procedure or post-procedure.

### Apheresis cell yields

**Fig. 4:**
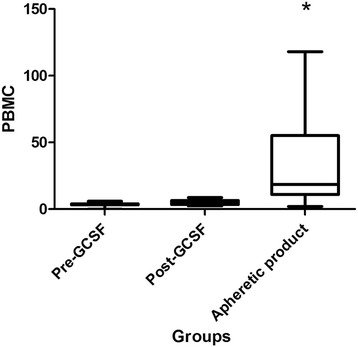
Numbers of Peripheral blood mononuclear cells (PBMC) in whole blood before (Pre-GCSF) (*n* = 8) and after administration of G-CSF (Post-GCSF) (*n* = 8) and in the apheretic product (*n* = 7). *Significantly different from Pre- and Post- GCSF values. *P* < 0.05. Values ×10^6^/ml blood

The final collection volume was approximately 35 ml in all animals. Following apheresis WBC concentrations had risen significantly from a pre-apheresis value of 16.1 ± 5.03 × 10^6^/ml to 38.5 ± 27.6 × 10^6^/ml and RBC concentration had significantly decreased from 6.4 ± 1.6 × 10^6^/ml to 1.9 ± 1.0 × 10^6^/ml. When individual cell types were quantified, the concentration of neutrophil in the apheretic product was reduced pre-apheresis (10.5 ± 4.2 × 10^6^/ml compared to post-apheresis of 6.4 ± 4) (Fig. 3). However, a significant increase in PBMC was seen. When all 7 animals were included in the analysis, including the non-responder, PBMC concentrations rose from 5.2 ± 1.8 × 10^6^/ml pre-apheresis to 27.5 ± 23.9 × 10^6^/ml post-apheresis. (Fig. 4).

**Fig. 5:**
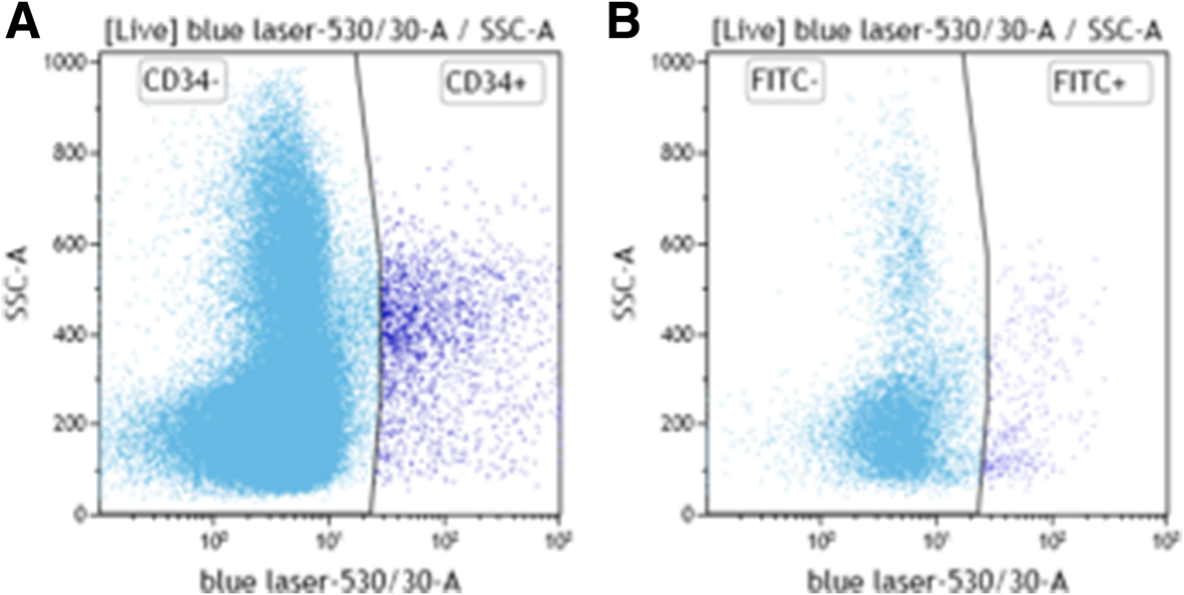
**a** Representative light-scatter (SSC-A) vs fluorescence (CD34-FITC) plot of CD34 expression on cells from ovine apheresis samples using two-colour flow cytometry. **b** IgG control

Using flow cytometry, the number of CD34+ and CD45+ cells in the apheresis product was measured. In the apheretic product 2.71 ± 2.3% of nucleated cells were CD34 + ve (Fig. 5.)

## Discussion

This report documents for the first time the successful concentration and collection of PBMC in sheep using automated apheresis and demonstrates that the procedure is a clinically safe method of obtaining a cell product enriched for PBMC containing CD34+ cells that did not pose significant technical difficulties.

Venous access was obtained via the jugular vein with the cephalic vein acting as the return portal. The apheresis machine was primed with saline rather than pre-collected blood as recommended in paediatric patients and small animal apheresis [9, 16]. In these smaller patients, priming with blood is required for successful apheresis as the extracorporeal circulating blood is a significant percentage of blood volume. In this study the sheep were sufficiently large (40-42 kg) that the haemodilution caused by the addition of saline to the circulation at the commencement of the cellular collection was not significant.

In 1/8 animals rapid repeated clotting of blood led to abandonment of the procedure in that individual. In 2 other animals clotting at the start of the procedure necessitated rapid placing of alternate catheters and/or flushing of tubing with heparin to remove blood clots before the pump could draw in the blood with sufficient pressure for effective centrifugation. The reason for such vigorous coagulation in 3/8 animals is not known. A study on interspecies difference in coagulation profiles reported that sheep had a similar clotting time and endogenous thrombin potential to humans [17] and so the response to the initial connection to the Cobe Spectra could not have been predicted. The appearance of clots coincided with the initial pressure from the machine within the vein to draw blood from the animal. Clotting is more likely at the beginning of the procedure as this is when the least amount of anticoagulant is distributed systemically. We recommend that particular attention is paid to the placing of the withdrawal portal in sheep undergoing apheresis and that all catheters/cannulas/tubing are primed with heparin prior to drawing blood.

In order to increase the numbers of circulating WBC containing the desired PBMC fraction, G-CSF was administered to the sheep prior to apheresis. G-CSF is a haematopoetic cytokine produced primarily by bone marrow stromal cells that acts, at higher doses, by stimulating the differentiation of several haematopoetic progenitors including CD34+ cells. In this study we administered 5 mg/kg rhG-CSF subcutaneously, daily for 3 days prior to apheresis. In a previous report this was the optimum dose for mobilisation of ovine WBC [13] and gave a peak response on day 3 of mobilisation, after which time, the total WBC dropped markedly. In this study, the sheep had a starting WBC count of 4.45 × 10^6^/ml, which rose to a post-GCSF value of approximately 13.5 × 10^6^/ml) [13]. These blood results mirror very closely the values we report in this paper and, coupled with the observation that rhG-CSF did not cause any adverse clinical effects in any of our sheep, support the use of commercially available rhG-CSF for the mobilisation of sheep WBC cells in the circulation. Of the 8 sheep treated with rhG-CSF one animal did not show an increase in WBC. In the other publication that reports the use of rhG-CSF in sheep, 1/3 animals did not respond. It has been reported that G-CSF based mobilisation regimes have a 5–30% failure rate among healthy individuals [18], with various factors being associated with a poor response to G-CSF in human patients, including age of patient, type of G-CSF used and prior exposure to other drugs. The reasons for poor mobilisation in animals are unknown, although they are likely to be similar.

Stimulation of the bone marrow using G-CSF and subsequent apheresis lead to a significant increase in WBC numbers and a significant decrease in RBC numbers in the apheretic product compared to unstimulated peripheral blood and peripheral blood obtained immediately prior to the apheresis technique. However, we did not find that the post-G-CSF WBC concentration was a predictor of apheretic product WBC concentration as has been reported in the dog [9]. Analysis of the cell types contributing to the increase in WBC numbers in the sheep reported here showed that neutrophils and PBMC were the cell types that were significantly increased in the apheretic product.

The PBMC fraction of blood is of particular interest in regenerative medicine as it contains HSC and progenitor cell populations. Whilst these progenitor cells have traditionally been used to treat malignant and non malignant haematopoetic disorders [5], they have also been used in regenerative medicine strategies to treat disorders of other body systems, including orthopaedic disease [6, 19] However, the identification of stem and progenitor cell populations in peripheral blood and apheretic products in veterinary species is not straightforward, due both to potential differences in stem cell CD designation and function between species and the availability of suitable reagents to characterise CD markers. In man, there is a recognised population of CD34 + ve cells that have stem/progenitor potential and are used for clinical transplantation [14]. In the sheep CD34 + ve cells have also been shown to have haematopoetic potential [20] and so CD34 appears to be an appropriate marker in this species, as well as man, for HSC. In this study, using flow cytometry and a sheep specific anti-CD34 antibody [14], we have shown that, 2.7% of the apheretic product was CD34 + ve. This figure is similar to the 1.5% reported in other studies [21] and supports our methodology.

## Conclusions

In conclusion this study has demonstrated that apheresis can be used in the sheep safely and without risk to the animal. The administration of G-CSF and subsequent apheresis provides a neutrophil and PBMC rich collection product containing > 2% of CD34 + ve cells that can be used for further in vivo or in vitro experimental investigations.

## Abbreviations

ACD-A: Acid-citrate-dextrose A
CBC: Complete blood counts
CD: Cluster designation
EDTA: Ethylenediaminetetraacetic acid
FITC: Fluorescein isothiocyanate
GCSF: Granulocyte colony stimulating factor
HSC: Haematopoetic stem cells
IgG: Immunoglobulin G
MSC: Mesenchymal stromal cells
PBMC: Peripheral blood mononuclear cell fraction
PBPC: Peripheral blood progenitor cell
RBC: Red blood cell
WBC: White blood cell
